# Minimally invasive mitral valve surgery after failed transcatheter mitral valve repair in an intermediate-risk cohort

**DOI:** 10.1093/icvts/ivac163

**Published:** 2022-06-17

**Authors:** Serdar Akansel, Markus Kofler, Karel M Van Praet, Axel Unbehaun, Simon H Sündermann, Stephan Jacobs, Volkmar Falk, Jörg Kempfert

**Affiliations:** German Heart Center Berlin, Department of Cardiothoracic and Vascular Surgery, Berlin, Germany; German Heart Center Berlin, Department of Cardiothoracic and Vascular Surgery, Berlin, Germany; DZHK (German Center for Cardiovascular Research), Partner Site, Berlin, Germany; German Heart Center Berlin, Department of Cardiothoracic and Vascular Surgery, Berlin, Germany; DZHK (German Center for Cardiovascular Research), Partner Site, Berlin, Germany; German Heart Center Berlin, Department of Cardiothoracic and Vascular Surgery, Berlin, Germany; DZHK (German Center for Cardiovascular Research), Partner Site, Berlin, Germany; German Heart Center Berlin, Department of Cardiothoracic and Vascular Surgery, Berlin, Germany; DZHK (German Center for Cardiovascular Research), Partner Site, Berlin, Germany; Charité – Universitätsmedizin Berlin, Department of Cardiovascular Surgery, Berlin, Germany; German Heart Center Berlin, Department of Cardiothoracic and Vascular Surgery, Berlin, Germany; DZHK (German Center for Cardiovascular Research), Partner Site, Berlin, Germany; German Heart Center Berlin, Department of Cardiothoracic and Vascular Surgery, Berlin, Germany; DZHK (German Center for Cardiovascular Research), Partner Site, Berlin, Germany; Charité – Universitätsmedizin Berlin, Department of Cardiovascular Surgery, Berlin, Germany; ETH Zurich, Department of Health Sciences and Technology, Zürich, Switzerland; German Heart Center Berlin, Department of Cardiothoracic and Vascular Surgery, Berlin, Germany; DZHK (German Center for Cardiovascular Research), Partner Site, Berlin, Germany

**Keywords:** Transcatheter mitral valve repair, Mitral repair, MitraClip, Transcatheter edge-to-edge repair, Transcatheter direct annuloplasty

## Abstract

**OBJECTIVES:**

Although clinical experience with transcatheter mitral valve interventions is rapidly increasing, there is still a lack of evidence regarding surgical treatment options for the management of recurrent mitral regurgitation (MR). This study provides guidance for a minimally invasive surgical approach following failed transcatheter mitral valve repair, which is based on the underlying mitral valve (MV) pathology and the type of intervention.

**METHODS:**

A total of 46 patients who underwent minimally invasive MV surgery due to recurrent or residual MR after transcatheter edge-to-edge repair or direct interventional annuloplasty between October 2014 and March 2021 were included.

**RESULTS:**

The median age of the patients was 78 [interquartile range, 71–82] years and the EuroSCORE II was 4.41 [interquartile range, 2.66–6.55]. At the index procedure, edge-to-edge repair had been performed in 45 (97.8%) patients and direct annuloplasty in 1 patient. All patients with functional MR at the index procedure (n = 36) underwent MV replacement. Of the patients with degenerative MR (n = 10), 5 patients were eligible for MV repair after removal of the MitraClip. The 1-year survival following surgical treatment was 81.3% and 75.0% in patients with functional and degenerative MR, respectively. No residual MR greater than mild during follow-up was observed in patients who underwent MV repair.

**CONCLUSIONS:**

Minimally invasive surgery following failed transcatheter mitral valve repair is feasible and safe, with promising midterm survival. The surgical management should be tailored to the underlying valve pathology at the index procedure, the extent of damage of the MV leaflets and the type of previous intervention.

**Figure:**
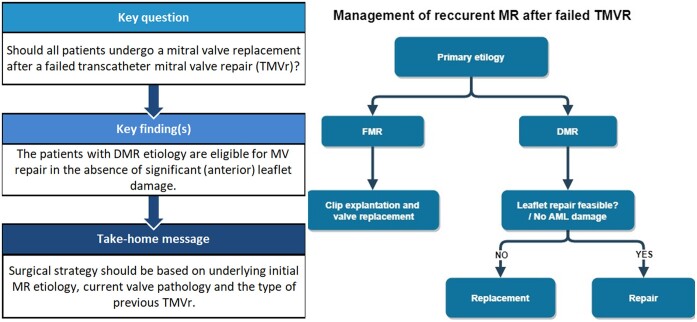

##  INTRODUCTION

Transcatheter mitral valve repair (TMVr) has evolved as an alternative therapeutic option to mitral valve surgery for high surgical risk or inoperable patients suffering from mitral regurgitation (MR). TMVr is indicated in symptomatic patients who are eligible according to echocardiographic criteria, at high surgical risk and for whom the procedure is not considered futile (class IIb recommendation) [1–3]. Although several transcatheter repair devices are commercially available, the largest experience has been gathered with the MitraClip system (Abbott Vascular, Menlo Park, CA, USA), which is based on the concept of the Alfieri technique [edge-to-edge (E2E) repair] [3]. The use of the MitraClip to restore leaflet coaptation has been mainly adopted for patients with functional mitral regurgitation (FMR).

Its safety and feasibility have been evaluated in numerous multicentre randomized controlled trials and large registries, such as the EVEREST I and EVEREST II trials (Endovascular Valve Edge-to-Edge Repair Study), the German Transcatheter Mitral Valve Interventions (TRAMI) registry, COAPT (Cardiovascular Outcomes Assessment of the Mitra Clip Percutaneous Therapy) and MITRA-FR trials [2, 4–7]. Given the long-term data of transcatheter E2E repair, the need for additional surgical procedures for the treatment of recurrent or residual MR is not negligible [2, 5, 8].

The number of patients with recurrent MR is increasing proportionally with the use of TMVr, and surgical reoperation may be much more challenging compared to native valve surgery. Severe fibrosis surrounding the device and leaflet damage may complicate device removal and the following valve operation, particularly when mitral valve repair is desired. The evidence regarding the optimal surgical treatment strategy for these patients is still limited, especially for a minimally invasive access [9, 10].

Therefore, our goal was to summarize our results and our clinical experience with minimally invasive mitral valve surgery (MI-MVS) following failed TMVr.

## PATIENTS AND METHODS

### Ethical statement

The study was approved by the institutional ethical committee (Charité Ethical Committee, Berlin; approval number EA4/041/21). Signed informed consent forms were obtained from all individuals. Surveillance information was obtained from the database of the Ministry of the Interior Residential Office. Demographic and other patient-related data were retrospectively obtained from electronic medical records.

### Patient population

From October 2014 to March 2021, a total of 46 consecutive patients undergoing MI-MVS due to recurrent or residual MR following TMVr (E2E repair or direct annuloplasty) were included in the study. Additional patients (*n* = 23) treated with a full sternotomy were not considered for the analysis (Supplementary Table 1). The main reasons for a non-minimally invasive approach were the necessity to perform concomitant procedures and anatomical factors such as severe mitral annular calcification, hostile chest (i.e. status post radiation) or cardiogenic shock.

**Figure 1:**
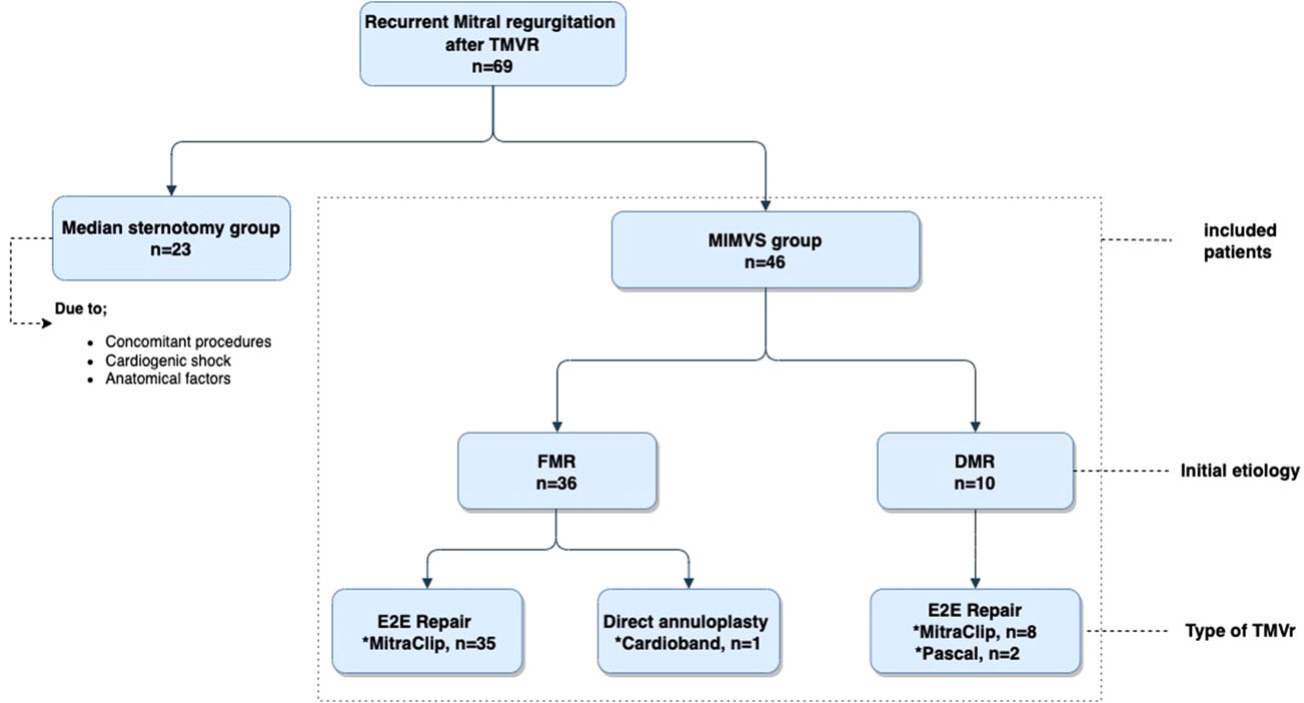
Flowchart showing study population. DMR: degenerative mitral regurgitation; E2E: edge-to-edge; FMR: functional mitral regurgitation; MIMVS: minimally invasive mitral valve surgery; TMVr: transcatheter mitral valve repair.

All patients were treated in accordance with respective European Society of Cardiology/European Association for Cardio-Thoracic Surgery guidelines [11, 12]. Acute procedural success for TMVr was defined as less than or equal to MR grade 2. Results were evaluated for 2 subgroups based on the underlying MR aetiology at the time of the index procedure (FMR and DMR) (Fig. 1).

### Patient characteristics

Baseline characteristics are presented in Table 1. At the time of MV surgery, 32 (69.5%) patients presented with progressive heart failure symptoms in New York Heart Association functional class III; 7 (15.2%) were in functional class IV. The median left ventricular ejection fraction was 55 [interquartile range (IQR), 40–55]. Ten patients (21.7%) had previous cardiac surgery including coronary artery bypass grafting (*n* = 7), aortic valve replacement (*n* = 2) and replacement of the ascending aorta (*n* = 1). Three patients were operated on urgently due to cardiac decompensation.

**Table 1:** Baseline characteristics

	All, n = 46	FMR, n = 36	DMR, n = 10
Age (years), median (IQR)	77.5 [71-82]	78,5 [71-82]	73,5 [67-82]
LVEF (%), median (IQR)	55 [40-55]	55 [40-55]	52 [32-55]
EuroSCORE II, median (IQR)	4.41 [2.65-6.62]	4.41 [2.70-6.89]	3.78 [2.01-5.97]
STS PROM score, median (IQR)	4.79 [2.88-7.28]	4.99 [3.33-7.07]	3.83 [2.09-7.77]
Male, n(%)	19 (41.3)	11 (30.5)	8 (80.0)
NYHA functional class, n(%)			
II	7 (15.2)	3 (8.3)	4 (40.0)
III	32 (69.5)	29 (80.5)	3 (30.0)
IV	7 (15.2)	4 (11.1)	3 (30.0)
HT, n (%)	35 (76.0)	29 (80.5)	6 (60.0)
DM, n (%)	8 (17.3)	8 (22.2)	0 (0)
Hyperlipidaemia, n(%)	17 (36.9)	14 (38.8)	3 (30.0)
CAD, n(%)	22 (47.8)	18 (50.0)	4 (40.0)
CRF, n(%)	29 (63.0)	24 (66.6)	5 (50.0)
Previous cardiac surgery, n (%)	10 (21.7)	10 (27.7)	0 (0)
CABG	7 (15.2)	7 (19.4)	0 (0)
AVR	2 (4.3)	2 (5.5)	0 (0)
Ascending aorta replacement	1 (2.1)	1 (2.7)	0 (0)

AVR: aortic valve replacement; CABG: coronary artery bypass graft; CAD: coronary artery disease; CRF: chronic renal failure; DM: diabetes mellitus; DMR: degenerative mitral regurgitation; FMR: functional mitral regurgitation; HT: hypertension; IQR: interquartile range; LVEF: left ventricular ejection fraction; NYHA: New York Heart Association; STS PROM: Society of Thoracic Surgeons predicted risk of mortality.

The median Society of Thoracic Surgery predicted risk of mortality score and European System for Cardiac Operative Risk Evaluation II (EuroSCORE II) were 4.79 [IQR, 2.88–7.28] and 4.41 [IQR, 2.66–6.55], respectively.

### Index procedure

The index TMVr procedures were direct annuloplasty with the Cardioband system in 1 patient and E2E repair in 45 patients (Pascal: *n* = 2 and MitraClip: *n* = 43). Three patients in the MitraClip group did not have a clip implanted due to unsuccessful attempts, and 3 other patients had undergone repeated clip implants because of recurrent MR. Detailed information regarding the index procedures is provided in Table 2. Only 4 patients included in the study underwent TMVr at our centre. The rest (*n* = 42) were referred to our centre for MVS from centres where TMVr was performed or followed.

**Table 2:** Data regarding index transcatheter procedures

	All, n = 46	FMR, n = 36	DMR, n = 10
TMVr, n(%)			
E2E repair	45 (97.8)	35 (97.2)	10 (100)
MitraClip	43 (93.4)	35 (97.2)	8 (80.0)
Pascal	2 (4.3)	0 (0)	2 (20.0)
Direct annuloplasty	1 (2.1)	1 (2.7)	0 (0)
No clip implanted, n(%)	3 (6.5)	3 (8.3)	0 (0)
Number of implanted clips, mean (SD)	1.83 (0.79)	1.78 (0.83)	1.88 (0.66)
Location of implanted clips, n(%)			
A1-P1	3 (6.5)	3 (8.3)	0
A2-P2	40 (86.9)	31 (86.1)	9 (90.0)
A3-P3	6 (13.0)	2 (5.6)	4 (40.0)
Time to surgery (day), median (IQR)			
With initial procedural success	211 [133-625]	438 [179.5-829.5]	133 [88-188]
Without initial procedural success	72.5 [21-367]	91 [28-647]	2 [n/a]

DMR: degenerative mitral regurgitation; E2E: edge-to-edge; FMR: functional mitral regurgitation; IQR: interquartile range; SD: standard deviation; TMVr: transcatheter mitral valve repair.

### Statistical analyses

Continuous data are provided as mean ± standard deviation or median with interquartile range (IQR) according to their distribution. Categorical variables are presented as absolute numbers and percentages. Survival probability for patients with FMR and DMR is depicted using Kaplan–Meier curves. All statistical values and data are provided in accordance with statistical and data reporting guidelines of the European Journal of Cardio-Thoracic Surgery and Interactive CardioVascular and Thoracic Surgery and the Strengthening the Reporting of Observational Studies in Epidemiology (STROBE) statement: guidelines for reporting of observational studies [13, 14].

### Surgical management

All patients underwent MI-MVS through a right anterolateral thoracotomy, with either video-assisted or 3D fully endoscopic visualization. The operations were performed by experienced surgeons in specialized MI-MVS centres. Cardiopulmonary bypass was established by cannulation of the femoral artery and vein utilizing a percutaneous or cut-down technique, depending on the preference of the surgeon. Myocardial protection was achieved by mild systemic hypothermia and antegrade infusion of cold Bretschneider’s cardioplegia (Custodiol) following cross-clamping.

**Figure 2:**
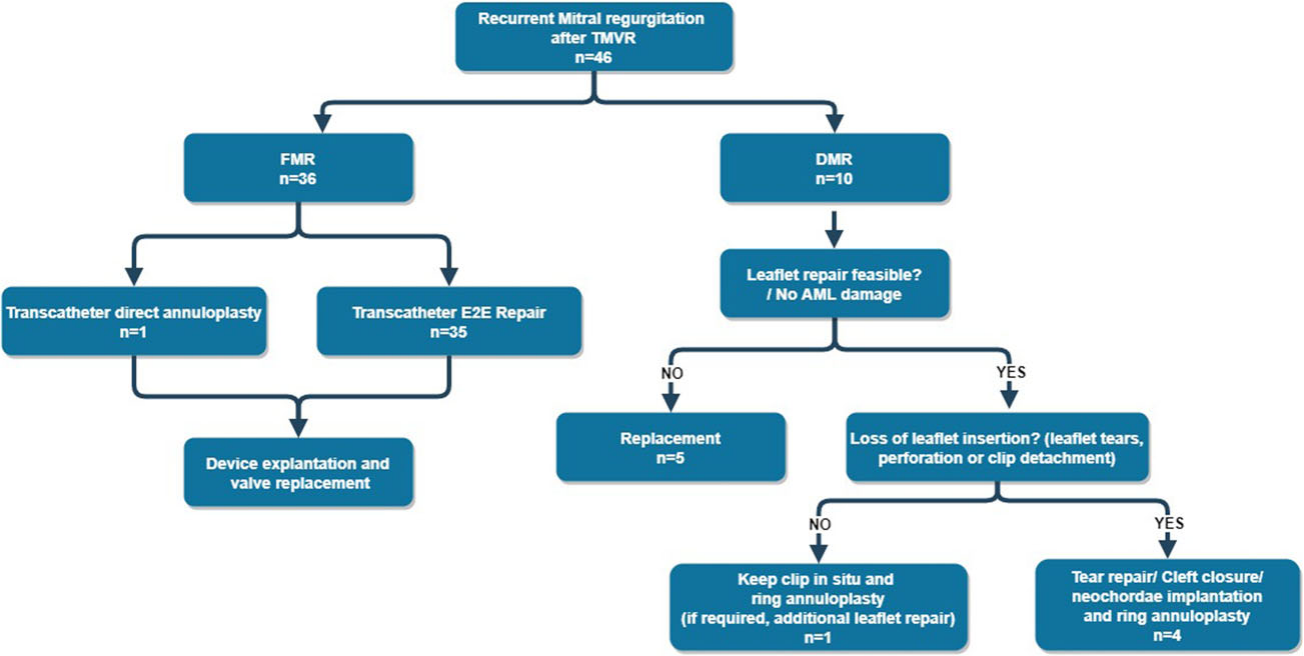
Surgical options regarding different clinical scenarios. Numbers represent the heading regarding surgical options in the surgical management section. AML: anterior mitral leaflet; DMR: degenerative mitral regurgitation; FMR: functional mitral regurgitation; MIMVS: minimally invasive mitral valve surgery; TMVr: transcatheter mitral valve repair.

Surgical management to troubleshoot the recurrent MR was determined by our interdisciplinary heart team based on the primary aetiology of the MR, the presence of the loss of leaflet insertion (LLI) and anterior leaflet damage and the feasibility of leaflet repair (Fig. 2).

#### Mitral valve replacement in patients with functional valve regurgitation

**Figure 3:**
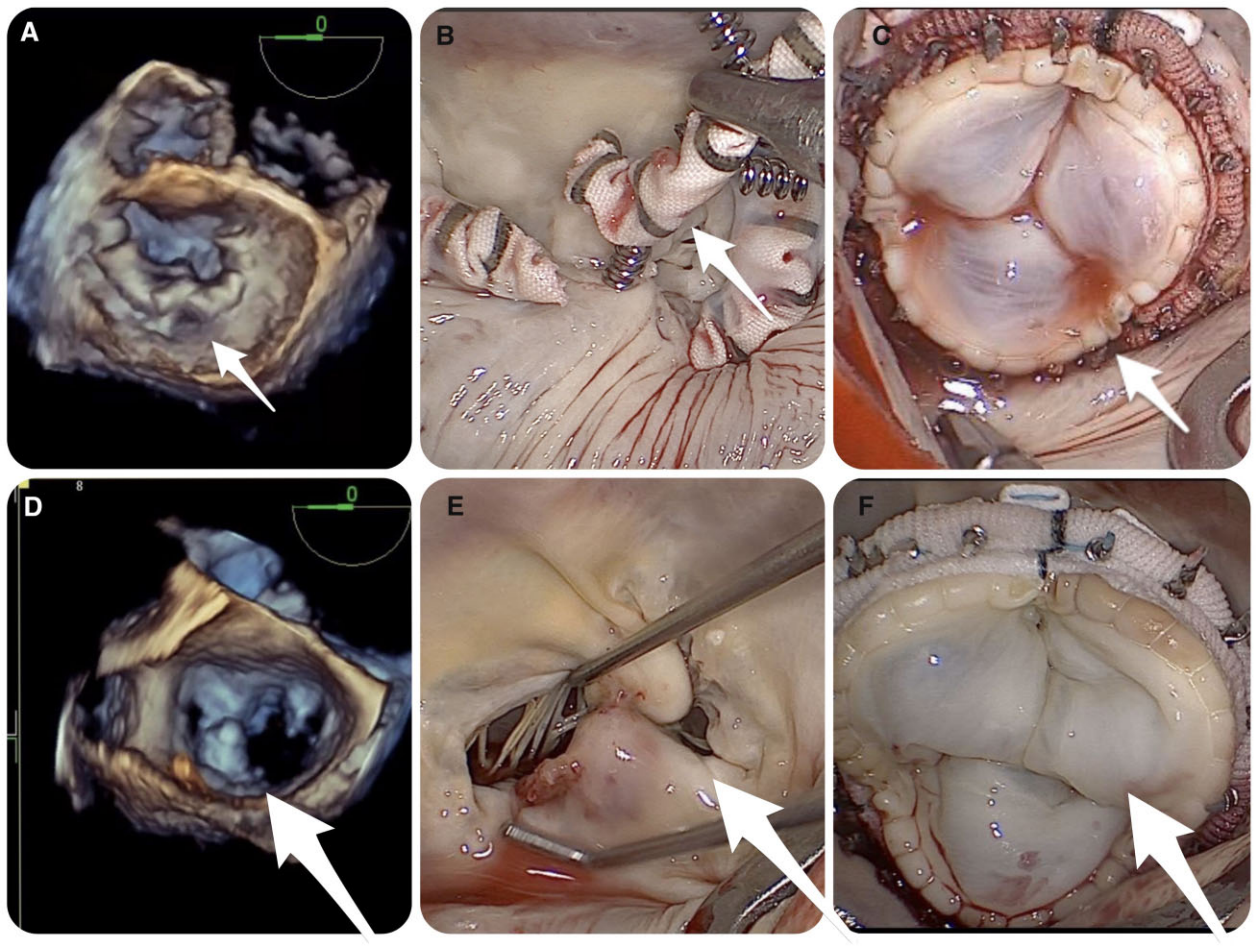
(A) Explantation of a Cardioband device: Perioperative transoesophageal echocardiography showing partial detachment of the device; **(B, C)** explantation with the cut-and-unscrew technique and valve replacement. **(D)** Perioperative transoesophageal echocardiography showing a MitraClip in place and annular dilatation; **(E, F)** mitral valve replacement with a biological prosthesis following removal of the MitraClip.

One patient referred after a Cardioband implant had severe MR due to partial detachment of the device from the posterior annulus at P2. Highly endothelialized parts of the device were dissected from the surrounding annular tissue. Special caution was applied not to damage any adjacent structures. The device was removed by applying a “cut and unscrew” technique. The Cardioband was cut between the anchors. Then, it was unscrewed by counterclockwise rotation (Fig. 3A). Given the low likelihood of successful repair and the high risk of recurrent MR, MV replacement was the surgical preference for this patient following removal of the device with the cut-and-unscrew technique, which has been described in detail elsewhere [15].

Within the group of patients who had an E2E repair due to FMR, 23 patients returned with pure MR, 1 patient had pure mitral stenosis and 10 patients had combined mitral regurgitation and stenosis. One patient underwent MV surgery 46 months after the index procedure due to mitral valve endocarditis. Detailed information regarding indications for MV surgery is provided in Table 3. In this group, LLI was observed in 15 patients. The main reason for recurrent or residual MR in patients without LLI was progression of the underlying disease and an unmet need for annuloplasty at the time of index TMVr.

**Table 3:** Indications for mitral valve surgery after transcatheter mitral valve repair

	All, n = 46	FMR, n = 36	DMR, n = 10
Urgency, n (%)	6 (13.0)	3 (8.3)	3 (30.0)
Mitral regurgitation, n (%)			
Recurrent	21 (45.6)	16 (44.4)	5 (50.0)
Residual	12 (26.0)	8 (22.2)	4 (40.0)
Mitral stenosis, n (%)	1 (2.1)	1 (2.7)	0 (0)
Mix of MR and stenosis, n (%)	11 (23.9)	10 (27.7)	1 (10.0)
MV endocarditis, n (%)	1 (2.1)	1 (2.7)	0 (0)
LLI, n (%)	24 (52.1)	15 (41.6)	9 (90.0)
Partial detachment	14 (23.3)	11 (30.5)	3 (30.0)
Embolization	2 (4.3)	0 (0)	2 (20.0)
Tear of leaflet	8 (17.3)	4 (11.1)	4 (40.0)

DMR: degenerative mitral regurgitation; FMR: functional mitral regurgitation; LLI: loss of leaflet insertion; MR: mitral regurgitation; MV: mitral valve.

The surgical choice of our centre was MV replacement for the management of residual or recurrent FMR. Accordingly, in these patients, mitral valve replacement with a biological prosthesis was performed after clip removal, with preservation of chordae tendineae to preserve the atrioventricular conjunction **(**Fig. 3B**).** Surgical details are provided in Table 4.

**Table 4:** Surgical details

	All, n = 46	FMR, n = 36	DMR, n = 10
MV repair, n (%)	5 (10.8)	0 (0)	5 (50.0)
Neochordae implantation	4 (8.6)	0 (0)	4 (40.0)
Ring annuloplasty	5 (10.8)	0 (0)	5 (50.0)
Cleft closure	4 (10.8)	0 (0)	4 (40.0)
MV replacement (with biological prosthesis), n (%)	41 (89.1)	36 (100)	5 (50.0)
Concomitant procedures, n (%)			
TVR	11 (23.9)	9 (25.0)	2 (20.0)
ASD closure	19 (41.3)	14 (38.8)	5 (50.0)
Cryoablation	15 (32.6)	12 (33.3)	3 (30.0)
In-hospital deaths, n(%)	3 (6.5)	2 (5.5)	1 (10.0)
ICU length of stay (h), median (IQR)	72 [26.0-149.5]	60 [25.2-123.7]	120 [43.2-232.5]
Hospital length of stay (days), median(IQR)	12 [8.0-17.0]	12 [8.0-14.7]	12 [7.7-23.0]

ASD: atrial septal defect; DMR: degenerative mitral regurgitation; FMR: functional mitral regurgitation; ICU: intensive care unit; IQR: interquartile range; MV: mitral valve; TVR: tricuspid valve repair.

#### Mitral repair or replacement in patients with degenerative mitral regurgitation

After removal of the MitraClip, the valve and leaflet tissue were evaluated for the feasibility of leaflet repair. Criteria that supported a decision towards valve replacement were defined as follows: The presence of major anterior mitral leaflet (AML) damage, non-repairable posterior mitral leaflet (PML) damage and calcification of leaflet tissue or insufficient PML tissue that did not allow for leaflet repair. Mitral valve repair was not feasible in 5 patients due to the presence of severe AML damage or irreparable PML injury that had occurred during the index procedure or in the postprocedural period. In these patients, MV replacement was performed with preservation of the chordae tendineae. One of these patients underwent MV replacement after an unsuccessful repair attempt.

**Figure 4:**
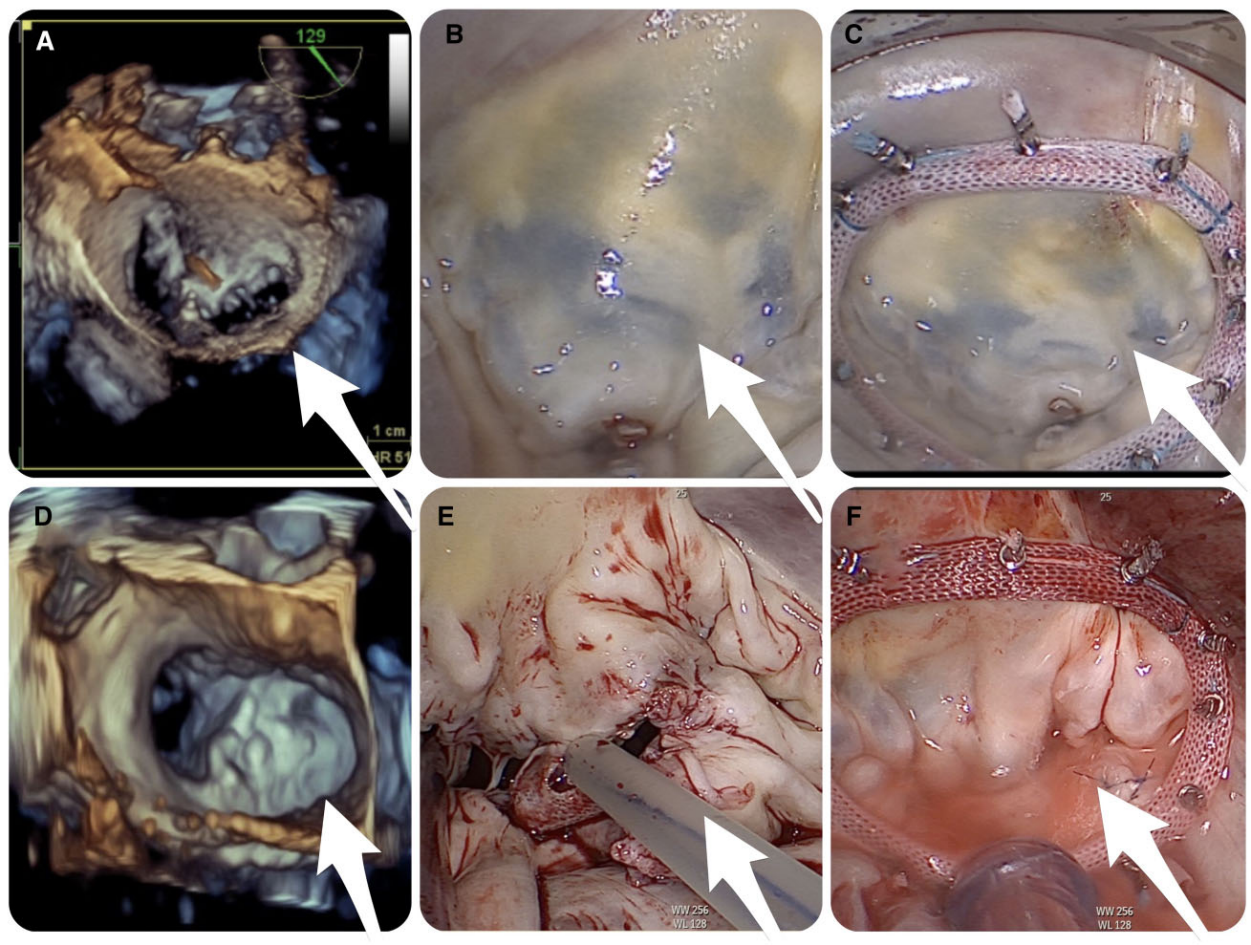
(A) A MitraClip device in the correct position and annular dilatation. **(B)** A saline test shows no leaflet prolapse, but regurgitation at the P1 and P2. **(C)** The clip was kept in situ and the annulus was supported with ring annuloplasty. **(D)** Perioperative transoesophageal echocardiography showing 2 MitraClip devices. **(E, F)** The surgical removal of the MitraClip device to maintain valvular integrity, facilitating subsequent neochordae implants, cleft closure and ring annuloplasty.

In the case of residual MR without LLI and without a stenotic component after a transcatheter E2E repair, one surgical approach is to keep the MitraClip in situ and to support the mitral valve complex with a ring annuloplasty to restore mitral coaptation. Among patients with DMR (*n* = 10), the MitraClip was retained in situ in 1 patient who had no LLI, and the mitral annulus was supported by ring annuloplasty (Fig. 4A).

In a case of LLI with no AML damage (*n* = 4), we have explanted the clips with sharp dissection of the leaflet edge captured by device arms, with the intention to preserve valve integrity for the purpose of possible MV repair. Our surgical technique in these cases has been described previously [16]. Briefly, following cleft closure and implanted artificial Gore-Tex chords (Gore-Tex; W.L. Gore & Associates, Inc, Newark, DE, USA), the repair was completed with semirigid rings. If necessary, the additional leaflet repair techniques such as cleft closure, neochordae implantation and sliding-plasty were performed (Fig. 4B).

### Data availability statement

The data underlying this article cannot be shared publicly due to the privacy of the individuals who participated in the study. The data will be shared on reasonable request to the corresponding author.

## RESULTS

### Outcome of patients with functional mitral regurgitation

**Figure 5:**
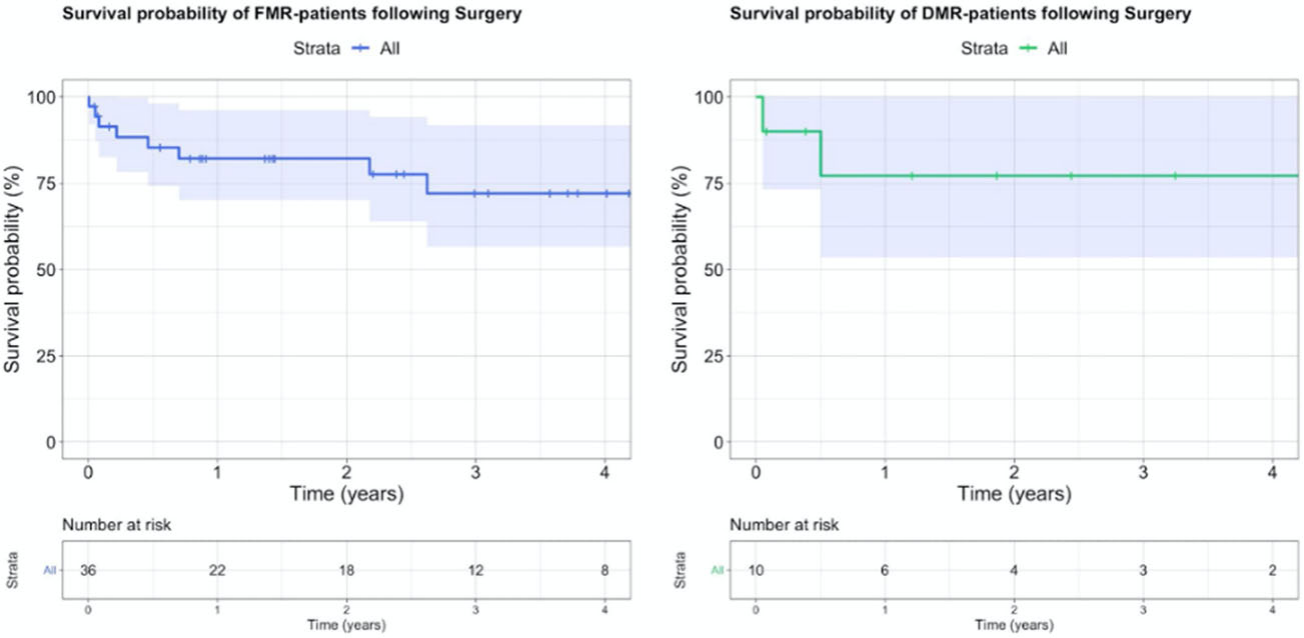
Survival probability of patients with functional mitral regurgitation and degenerative mitral regurgitation following surgery due to failed transcatheter mitral valve repair. DMR: degenerative mitral regurgitation; FMR: functional mitral regurgitation.

Two patients died in-hospital (5.5%). Of these patients, 1 died 20 days postoperatively of sepsis, multiple organ dysfunction syndrome and renal failure. The other patient died of ventricular fibrillation cardiac arrest 48 h after the operation. All other patients were discharged a median of 12 [IQR, 8–14.7] days after surgery, with no residual MR (Table 4). Overall survival was 81.25% at the 1-year follow-up (Fig. 5).

### Outcome of patients with degenerative mitral regurgitation

One patient (10%) who received an MV replacement died 18 days after surgery of multiple organ dysfunction syndrome resulting in septic shock. There was no perioperative myocardial infarctions, strokes or rethoracotomies. Predischarge transthoracic echocardiography revealed mild (*n* = 2) or no (*n* = 8) residual MR. After the 1-year follow-up, overall survival was 75% (Fig. 5).

## DISCUSSION

This study summarizes our clinical experience with 46 consecutive patients undergoing MI-MVS after failed TMVr and suggests an algorithm to select surgical options based on the underlying MV pathology and the type of previous intervention.

The lessons learned and the main messages from the present study can be summarized as follows: (1) MI-MVS can be safely performed with very favourable early-term outcomes and low MR recurrence in patients after failed TMVr. (2) Our decision-making strategy is based on underlying initial MR aetiology, existing valve pathology and the type of previous TMVr. (3) Our surgical strategy is to perform MV replacement in patients with FMR and MV repair in patients with DMR without significant leaflet damage and if repair is feasible. It should be highlighted that this patient group typically presents with a high surgical risk profile and had been judged “unsuited” for cardiac surgery by the heart team at the time of the index procedure. In our cohort, 21.7% were redo procedures but still could be managed using a minimally invasive approach.

The fact that the safety and feasibility of the MitraClip were confirmed in EVEREST II, TRAMI Registry, COAPT and MITRA-FR trials makes TMVr an increasingly popular treatment option for high-risk patients [2, 5–7]. The promising results have encouraged researchers and companies to develop new transcatheter E2E repair devices such as Pascal or a transcatheter annuloplasty device, the Carillon (Cardiac Dimensions, Kirkland, WA, USA), Mitralign (Mitralign, Inc, Tewksbury, MA USA) or Cardioband. However, given the inferior long-term results regarding the efficacy of TMVr in patients with DMR, MV surgery still remains the gold standard for those who are not at high surgical risk [1]. Additionally, it is one of the bailout options after failed transcatheter attempts due to recurrence of MR. The 5-year results of the EVEREST II trial have demonstrated the superiority of surgery in freedom from death, reoperation for MV dysfunction and recurrence of severe MR [2]. The 1-year follow-up results of different trials and registries have shown that a surgical reintervention became necessary in 5.7% of patients in the EVEREST II, in 2.8% of patients in the TRAMI registry and in 6.3% of the patients in the ACCESS-EU trial [2, 5, 8]. A total of 3+ or 4+ MR was detected in 21.1% of patients at the 1-year follow-up in the ACCESS-EU trial [8].

The increasing numbers of patients with recurrent MR after failed TMVr have confronted surgeons with new challenges. The feasibility of surgical MV repair after TMVr has been recently evaluated in several studies, mostly in patients with predominantly degenerative MV diseases. Geidel and colleagues reported the infeasibility of MV repair in patients with 2 or more clip implants due to device-induced tissue damage [10]. Moreover, Glower *et al.* reported that the presence of anterior/bileaflet flail or prolapse significantly predicted MV replacement [17]. On the contrary, another series reported that clip explant procedures and encapsulation of clips did not affect the subsequent surgical management in patients undergoing MV repair [18, 19]. Similarly, Argenziano *et al.* demonstrated that MV repair was feasible in 21 of 32 patients with prior MitraClips and mostly with predominantly DMR [9]. However, the feasibility of MV repair was less than 10% in patients included in the CUTTING-EDGE Registry, which did not focus on the minimally invasive setting [20].

From our point of view, a new surgical strategy should be defined for patients undergoing MV surgery in the setting of prior TMVr. In patients with FMR, prognosis is dominated by underlying left ventricular remodelling, which necessitates a special management strategy for MV surgery in this group. A residual relevant MR may not be tolerated and may result in volume overload of the left ventricle. Moreover, the benefits of surgical repair in these cases are controversial [21, 22]. In patients with FMR with a prior TMVr, an unsuccessful surgical repair may result in undesirable outcomes. However, a high in-hospital mortality rate has been reported for patients with FMR undergoing MV replacement after failed TMVr [23]. In this context, it should be highlighted that the patients in our study with FMR may not adequately represent a classical cohort of patients with FMR due to a relatively high LVEF and the intermediate surgical risk profile of the patient group. However, when the FMR group was analysed in depth, the LVEF in patients with the ventricular type of FMR was lower than that in the atrial type of subgroup (Supplementary Table 2). Moreover, hospital deaths in the FMR group undergoing MI-MVS were much lower than those in patients with FMR undergoing a full sternotomy. However, it should be taken into account that the expected mortality was higher in patients who had full sternotomies because they had more comorbidities such as anatomical factors, cardiogenic shock or the need for concomitant cardiac surgery.

In the light of the preceding arguments, our decision-making strategy was based on the underlying MV pathology and the type of prior TMVr. This strategy is to perform MV replacement in patients with FMR and MV repair in patients with DMR if repair is feasible. Because the underlying pathology is often FMR in patients with prior transcatheter direct annuloplasty, the strategy was removal of the device and subsequent replacement of the MV.

In patients with DMR, our surgical strategy was based on the following criteria:

- If there was no LLI, the clip was kept in situ and the annulus was supported by ring annuloplasty. If necessary, additional leaflet repair procedures can be performed. This technique may largely reduce the risk of leaflet damage, which may preclude MV repair during removal of an intact clip, especially in the case of encapsulated clips.
- LLI was accepted as a criterion for clip removal. The subsequent strategy was based on the integrity of the AML and the feasibility of PML repair. Damage to the AML was defined as having a high risk of unsuccessful MV repair, as previously reported by Glower and colleagues [24]. Degenerative processes around the implanted clip, leaflet tear or rupture resulting in LLI have been reported as the most common factors limiting the feasibility of repair [25]. These structural and device-related pathologies also limit the feasibility of repeat TMVr. In our series, the patients with AML damage or non-repairable PML injury underwent MV replacement.

Unlike previous reports on MV surgery after failed interventions that were almost exclusively performed through a median sternotomy, our study is the first report summarizing the results of a systematic minimally invasive approach after TMVr. In previous studies, it has been reported that MI-MVS can be safely performed with minimal morbidity and mortality [26], especially in high-risk groups such as patients with left ventricular dysfunction [27], with previous cardiac surgery [28, 29] and those who are elderly [30]. Our favourable results suggest that MI-MVS is also a safe and effective procedure for patients undergoing MV surgery after failed TMVr, even in a redo setting.

## CONCLUSION

A minimally invasive approach for patients with recurrent MR after TMVr procedures is feasible but necessitates adjustment of the surgical strategies. Patients with FMR should undergo MV replacement due to the risk of recurrent MR after MV repair. Patients with DMR may be eligible for MV repair in the absence of significant leaflet damage. MI-MVS can be safely performed in patients after failed TMVr, with favourable outcome and low recurrence of MR in case of mitral valve repair.

## LIMITATIONS

We acknowledge that this study has some limitations. First, the design of the study is retrospective. Second, we report our single-centre experience with patients undergoing MI-MVS. Multicentre studies with larger patient groups are required. Third, most of patients had undergone a TMVr at another centre. This fact has limited our ability to obtain detailed information regarding the index procedures. Another limitation was the small number of patients undergoing MV repair, which limited our ability to perform statistical analyses regarding the surgical outcomes. The 95% confidence intervals were not adjusted for multiple comparisons, and inferences drawn from them may not be reproducible. Building on these limitations, further research is required to clarify which surgical strategy is the best option in patients undergoing MV surgery after a failed TMVr.

## FUNDING

No funding was provided for the present study.

## CONFLICT OF INTEREST STATEMENT

All authors meet all the criteria for authorship and declare that they have no competing interests. V. Falk declares that he has relevant financial activities outside the submitted work with the following commercial entities: Medtronic GmbH (Meerbusch, Germany), Biotronik SE & Co. (Berlin, Germany), Abbott GmbH & Co. KG (Wiesbaden, Germany), Boston Scientific (Ratingen, Germany), Edwards Lifesciences (Unterschleissheim, Germany), Berlin Heart (Berlin, Germany), Novartis Pharma GmbH (Nuremburg, Germany), JOTEC GmbH (Hechingen, Germany) and Zurich Heart (Zurich, Switzerland) in relation to educational grants (including travel support), fees for lectures and speeches, fees for professional consultation and research and study funds. J. Kempfert reports personal fees from Edwards, personal fees from LSI, outside the submitted work. The authors have no other relevant affiliations or financial involvements.

## AUTHOR CONTRIBUTIONS

**Serdar Akansel**: Conceptualization; Data curation; Formal analysis; Methodology; Writing—original draft. **Markus Kofler**: Conceptualization; Formal analysis; Methodology; Writing—review & editing. **Karel M. Van Praet**: Conceptualization; Data curation. **Axel Unbehaun**: Conceptualization; Supervision. **Simon H. Sündermann**: Conceptualization; Writing—review & editing. **Stephan Jacobs**: Conceptualization; Supervision. **Volkmar Falk**: Conceptualization; Supervision; Writing—review & editing. **Jörg Kempfert**: Conceptualization; Methodology; Supervision; Validation; Writing—review & editing.

## Supplementary Material

ivac163_Supplementary_Data

## Notes

The manuscript was presented as a visual abstract at the 35^th^ annual meeting of the European Association For Cardio-Thoracic Surgery

## ABBREVIATIONS

AML: Anterior mitral leaflet
DMR: Degenerative mitral regurgitation
E2E: Edge-to-edge
FMR: Functional mitral regurgitation
IQR: Interquartile range
LLI: Loss of leaflet insertion
MI-MVS: Minimally invasive mitral valve surgery
MR: Mitral regurgitation
MV: Mitral valve
PML: Posterior mitral leaflet
TMVr: Transcatheter mitral valve repair
