# Supplementary material for: Minimally invasive mitral valve surgery after failed transcatheter mitral valve repair in an intermediate-risk cohort
Source: Interact Cardiovasc Thorac Surg. 2022 Jun 17;35(2):ivac163. doi: 10.1093/icvts/ivac163 (PMC9270869; doi:10.1093/icvts/ivac163)
Supplement: ivac163_Supplementary_Data [file ivac163_supplementary_data.zip › Supplementary 2.docx]

**Table 1:** Patient characteristics in two subgroups in terms of atrial or ventricular type of FMR.

|  | **All, n=36** | **atrial, n=21** | **Ventricular, n=15** |
| --- | --- | --- | --- |
| Age (years), median (IQR) | 78,5 [71-82] | 79 [71-82.5] | 75 [71-82] |
| LVEF (%), median (IQR) | 55 [40-55] | 55 [53.5-60] | 35 [30-40] |
| LVEDD (mm), median (IQR) | 50 [45-61] | 48 [45-50] | 61 [53-66] |
| Euroscore II, median (IQR) | 4.41 [2.70-6.89] | 4.08 [2.87-9.15] | 4.75 [2.65-6.45] |
| STS Prom score, median (IQR) | 4.99 [3.33-7.07] | 4.64 [3.42-5.64] | 5.83 [3.24-8.35] |
| Hospital Mortality, n(%) | 2(6.5) | 0(0) | 2 (13.3) |
| One-year Survival (%) | 81.25 | 88.88 | 71.42 |

IQR: Interquartile range; LVEF: Left ventricular ejection fraction; LVEDD: Left ventricular enddiastolic diameter
