# Supplementary material for: Minimally invasive mitral valve surgery after failed transcatheter mitral valve repair in an intermediate-risk cohort
Source: Interact Cardiovasc Thorac Surg. 2022 Jun 17;35(2):ivac163. doi: 10.1093/icvts/ivac163 (PMC9270869; doi:10.1093/icvts/ivac163)
Supplement: ivac163_Supplementary_Data [file ivac163_supplementary_data.zip › Supplementary 1.docx]

**Table 2.** Patient characteristics and survival data in full sternotomy group.

|  | **All, n=23** | **FMR, n=18** | **DMR, n=5** |
| --- | --- | --- | --- |
| Age (years), median (IQR) | 76 [73-82] | 76 [68.75-81.25] | 84 [78-87] |
| LVEF (%), median (IQR) | 50 [35-55] | 47.5 [33.75-55] | 55 [50-62.5] |
| Euroscore II, median (IQR) | 6.51 [5.55-8.51] | 6.78 [5.64-8.77] | 6.09 [2.70-6.99] |
| STS Prom score, median (IQR) | 6.62 [5.22-8.22] | 6.78 [5.28-8.33] | 5.76 [3.27-9.11] |
| Hospital Mortality, n(%) | 6(26.0) | 5(27.7) | 1 (20.0) |
| One-year Survival (%) | 65.21 | 61.1 | 80.0 |

LVEF: Left ventricular ejection fraction; IQR: Interquartile range
